# Structure of *Staphylococcus aureus* adenylo­succinate lyase (PurB) and assessment of its potential as a target for structure-based inhibitor discovery

**DOI:** 10.1107/S0907444910020081

**Published:** 2010-07-09

**Authors:** Paul K. Fyfe, Alice Dawson, Marie-Theres Hutchison, Scott Cameron, William N. Hunter

**Affiliations:** a Division of Biological Chemistry and Drug Discovery, College of Life Sciences, University of Dundee, Dundee DD1 5EH, Scotland

**Keywords:** adenylosuccinate lyase, AMP, oxalate, purine biosynthesis, purine cycle

## Abstract

The medium-resolution structure of adenylosuccinate lyase (PurB) from the bacterial pathogen *Staphylococcus aureus* in complex with AMP is presented. Oxalate, which is likely to be an artifact of crystallization, has been modelled in the active site and occupies a position close to that where succinate is observed in orthologous structures. PurB catalyzes reactions that support the provision of purines and the control of AMP/fumarate levels. As such, the enzyme is predicted to be essential for the survival of *S. aureus* and to be a potential therapeutic target. Comparisons of this pathogen PurB with the enzyme from *Escherichia coli* are presented to allow discussion concerning the enzyme mechanism. Comparisons with human PurB suggest that the close similarity of the active sites would make it difficult to identify species-specific inhibitors for this enyme. However, there are differences in the way that the subunits are assembled into dimers. The distinct subunit–subunit interfaces may provide a potential area to target by exploiting the observation that creation of the enzyme active site is dependent on oligomerization.

## Abstract 2

The 2.5 Å resolution structure of *S. aureus* adenylosuccinate lyase is reported and compared with those of orthologues to assess its potential as a template for early stage drug discovery. AMP and a putative assignment of oxalate, the latter an artefact possibly arising from an impurity in the PEG used for crystallization, occupy the active site.

## 1. Introduction

**Figure 1:**
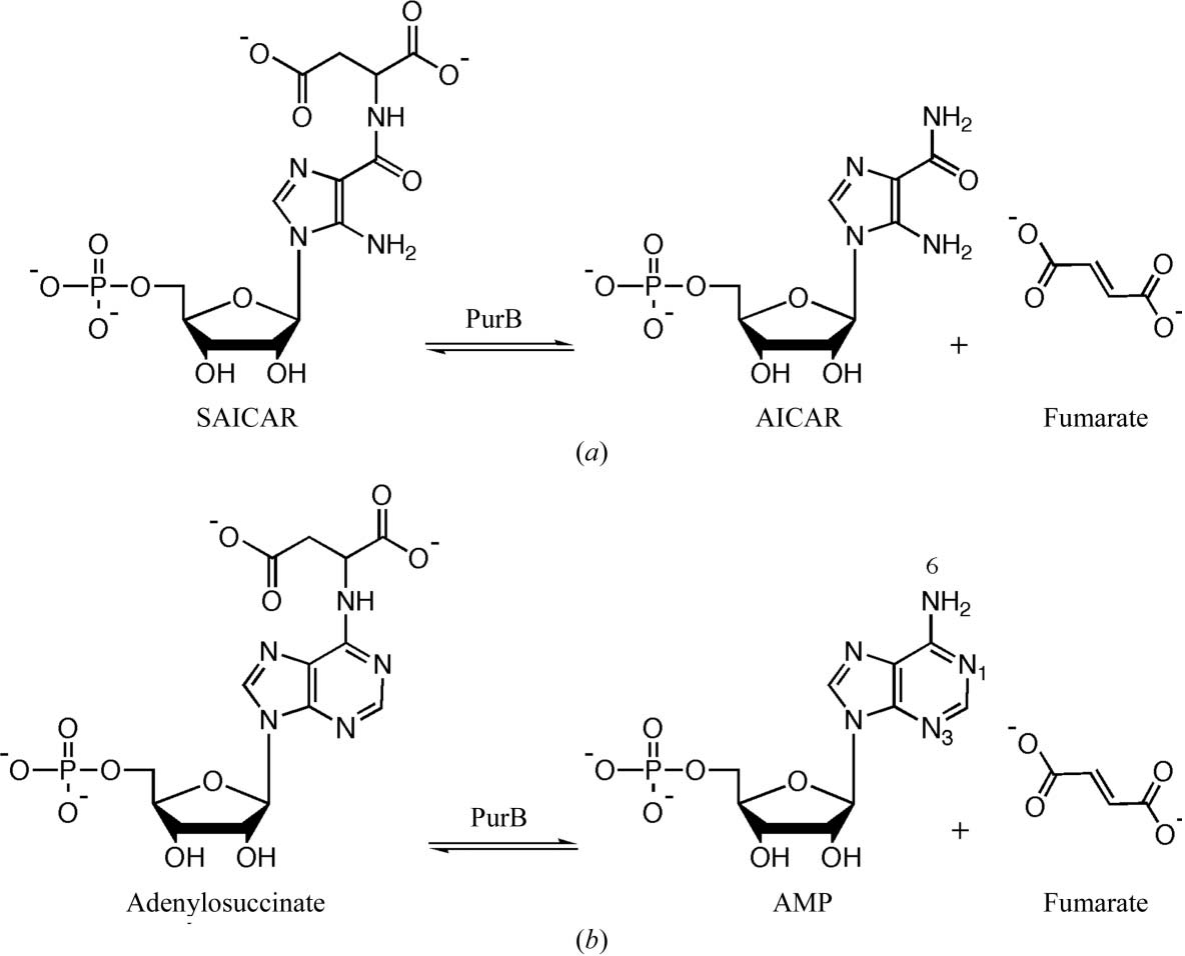
PurB catalyzes two reactions: the conversion of SAICAR to AICAR (*a*) and the conversion of adenylosuccinate to AMP (*b*). In each case fumarate is also produced.

The purine-biosynthetic pathway is ultimately responsible for the generation of inosine 5′-monophosphate from α-d-ribose-5-phosphate (Zhang *et al.*, 2008 ▶) and provides the essential purine nucleotides required for DNA replication and cell division. The pathway consists of ten enzyme-catalyzed steps in vertebrates (Buchanan & Hartman, 1959 ▶) and 11 in *Escherichia coli* (Mueller *et al.*, 1994 ▶). In this pathway, adenylosuccinate lyase (PurB; EC 4.3.2.2) catalyzes the breakdown of 5-aminoimidazole-4-(*N*-succinylcarboxamide) ribotide (SAICAR) to 5-aminoimidazole-4-carboxamide ribotide (AICAR) and fumarate (Fig. 1 ▶). PurB demonstrates dual substrate specificity and can also break down adenylo­succinate to adenosine monophosphate (AMP) and fumarate (Fig. 1 ▶); therefore, it also determines the levels of AMP and fumarate in the cell *via* the purine-nucleotide cycle. In humans, mutations in PurB and adenylosuccinate lyase deficiency have profound effects on the central nervous system (Spiegel *et al.*, 2006 ▶). It has been predicted on the basis of bioinformatics comparisons and metabolic reconstructions that the *purB* gene encodes an essential enzyme activity in *Staphylococcus aureus* (Heinemann *et al.*, 2005 ▶), although we note that it would be important to genetically and chemically validate this prediction. Selective inhibition of PurB offers the possibility of new therapies targeting a range of microbial infections and structural studies provide useful data to assess the potential of this protein for such early stage drug discovery (Hunter, 2009 ▶).

PurB is classified along with arginosuccinate lyase, l-­aspartase, class II fumarases and 3-carboxymuconate lactonase as a member of the aspartase/fumarase or β-elimination superfamily of enzymes (Fujii *et al.*, 2003 ▶). Crystal structures of PurB from a number of organisms have been determined. These include orthologues from the archaea *Pyrobaculum aerophilum* (Toth *et al.*, 2000 ▶) and *Thermotoga maritima* (Toth & Yeates, 2000 ▶), the eukaryotes *Caenorhabditis elegans* (PDB code 1yis; J. Symersky *et al.*, unpublished work), *Homo sapiens* (PDB code 2vd6; P. Stenmark *et al.*, unpublished work) and *Plasmodium vivax* (PDB code 2qga; Vedadi *et al.*, 2007 ▶) and the bacteria *E. coli* (Tsai *et al.*, 2007 ▶), *Bacillus anthracis* (PDB code 2pfm; V. M. Levdikov, E. V. Blagova, A. J. Wilkinson & K. S. Wilson, unpublished work), *B. subtilis* (Toth *et al.*, 2000 ▶) and *Legionella pneumophila* (PDB code 3bhg; C. Chang, H. Li, L. Freeman & A. Joachimiak, unpublished work).

We describe the construction of an efficient bacterial recombinant expression system, enzyme purification and crystallization protocols and report the crystal structure of PurB from *S. aureus* (*Sa*PurB) at 2.5 Å resolution. Our structure contains one of the reaction products, AMP, bound in the active site and a nearby strong feature of electron density has been modelled and successfully refined as oxalate. Comparisons with previously determined PurB structures are presented, concentrating on active-site ligand interactions and the mode of dimerization.

## 2. Methods

### 2.1. Cloning and sample preparation

The *S. aureus* *purB* gene was amplified from genomic DNA (American Type Culture Collection strain 35556, Laboratory of the Government Chemist Standards Office, UK) using the primers 5′-catatgATTGAACGCTATTCTCGAG and 5′-ctc­gagTTATGCTAATCCAGCGCG (the lower-case sequences correspond to the *Nde*I/*Xho*I restriction sites used for cloning). Following TOPO cloning (Invitrogen) of the PCR product, the gene was ligated into a pET15b (Novagen) expression vector modified to encode a tobacco etch virus (TEV) protease cleavage site in place of the thrombin protease cleavage site. This vector was transformed into *E. coli* BL21 (DE3) pLysS (Stratagene). Cultures were grown at room temperature in Luria–Bertani medium supplemented with 50 mg ml^−1^ ampicillin and 12 mg ml^−1^ chloramphenicol overnight, with 0.5 m*M* isopropyl β-d-1-thiogalactopyranoside added to induce gene expression, and the cells were harvested by centrifugation (3000*g* at 277 K for 30 min). The pellet was resuspended in 50 m*M* Tris–HCl pH 7.5, 250 m*M* NaCl, 20 m*M* imidazole and the cells were lysed using a French press. Cell debris was removed by centrifugation (40 000*g* at 277 K for 30 min).

*Sa*PurB was purified with a protocol commonly used in our laboratory (Dawson *et al.*, 2008 ▶). In summary, the first stage involved nickel-affinity chromatography on a 5 ml HisTrap column (GE Healthcare). A linear concentration gradient was applied to elute the product, which was dialyzed against 50 m*M* Tris–HCl, 250 m*M* NaCl; the His tag was then removed by incubation overnight with His-tagged TEV protease. The resulting mixture was applied onto the HisTrap column, which bound the cleaved His tag, the TEV protease and uncleaved *Sa*PurB. The *Sa*PurB from which the His tag had been cleaved was present in the flowthrough. Fractions were analyzed using SDS–PAGE and those containing *Sa*PurB were pooled. The protein was further purified by size-exclusion chromatography using a Superdex 200 26/60 column (GE Healthcare) equilibrated with 50 m*M* Tris–HCl, 250 m*M* NaCl pH 7.5. This column had previously been calibrated with molecular-weight standards: blue dextran (>2000 kDa), thyro­globulin (669 kDa), ferritin (440 kDa), aldolase (158 kDa), conalbumin (75 kDa), ovalbumin (43 kDa), carbonic anhydrase (29.5 kDa), ribonuclease A (13.7 kDa) and aprotinin (6.5 kDa) (GE Healthcare; data not shown). The high level of *Sa*PurB purity was confirmed by SDS–PAGE and matrix-assisted laser desorption/ionization–time-of-flight mass spectrometry. The sample was dialyzed into 20 m*M* Tris–HCl pH 7.5, 50 m*M* NaCl and concentrated using a Vivaspin 20 (Sartorius) to provide a stock solution for crystallization. A theoretical extinction coefficient of 59 485 *M* ^−1^ cm^−1^ at 280 nm was used to estimate the protein concentration (*PROTPARAM*; Gasteiger *et al.*, 2005 ▶); the theoretical mass of one subunit was estimated as 49.6 kDa.

*Sa*PurB was crystallized at 293 K by the hanging-drop vapour-diffusion method using 1 µl protein solution at a concentration of 11 mg ml^−1^ containing 25 m*M* AMP mixed with 1 µl reservoir consisting of 45% polyethylene glycol (PEG) 200, 0.2 *M* NaCl and 0.1 *M* sodium phosphate buffer pH 6.2. Orthorhombic blocks with minimum dimensions of 0.2 mm grew over 2–3 weeks. Crystals were first characterized in-house using a Rigaku MicroMax-007 rotating-anode X-ray generator coupled to an R-AXIS IV^++^ image-plate detector. The amount of PEG 200 in the mother liquor allowed crystals to be mounted directly from the drop without additional cryoprotection. Autoindexing revealed that the crystals were ortho­rhombic, with unit-cell lengths that were subsequently determined as *a* = 81.6, *b* = 121.3, *c* = 105.7 Å. Suitable crystals were stored in liquid N_2_ for use in data collection at the Diamond Light Source (Rutherford Appleton Laboratories, England).

### 2.2. X-ray data collection, processing, structure solution and refinement

Diffraction data were measured on beamline I04 of the Diamond Light Source using an ADSC Q315 CCD detector. Data were indexed and integrated using *MOSFLM* (Leslie, 2006 ▶) and scaled using *SCALA* (Evans, 2006 ▶); statistics are summarized in Table 1 ▶. The structure was solved by molecular replacement using the structure of adenylosuccinate lyase from *T. maritima* (PDB code 1c3c; Toth & Yeates, 2000 ▶) as the search model. This model shares approximately 50% sequence identity to *Sa*PurB. One molecule was located per asymmetric unit, giving a *Z* score of 21.1 using the program *Phaser* (McCoy *et al.*, 2007 ▶). Refinement was performed in *REFMAC*5 (Murshudov *et al.*, 1999 ▶) utilizing translation/libration/screw refinement and alternated with inspection of Fourier syntheses (2*F*_o_ − *F*_c_ and *F*_o_ − *F*_c_) and model manipulation together with ligand incorporation using *Coot* (Emsley & Cowtan, 2004 ▶). *MolProbity* (Chen *et al.*, 2010 ▶) was used to investigate model geometry in combination with the validation tools provided in *Coot*. Crystallographic statistics are presented in Table 1 ▶. Analyses of surface areas and inter­actions were made using the *PISA* service (Krissinel & Henrick, 2007 ▶) and figures were prepared with *PyMOL* (DeLano, 2002 ▶). Amino-acid sequence alignments were carried out using the program *MUSCLE* (Edgar, 2004 ▶). The coordinates and structure-factor data have been deposited in the Protein Data Bank with accession code 2x75.

## 3. Results and discussion

### 3.1. General comments

**Figure 2:**
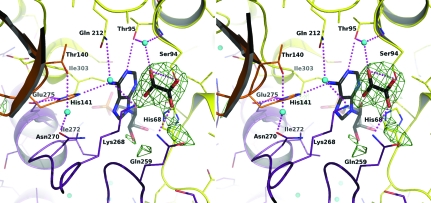
Stereoview of the OMIT map of oxalate and interactions in the active site. The OMIT *F*_o_ − *F*_c_ difference density map is shown as green chicken wire and contoured at 3σ. The protein main chain is shown in ribbon format coloured according to subunit: yellow, subunit *A*; orange, subunit *B*; purple, subunit *D*. Side chains are shown as sticks coloured red for O atoms, blue for N atoms, orange for P atoms and according to subunit as before for C atoms. The oxalate and AMP C positions are shown in black. Water molecules are depicted as cyan spheres. Dashed lines represent potential hydrogen-bonding interactions.

*Sa*PurB crystallizes in an orthorhombic system and the systematic absences indicated either space group *I*222 or *I*2_1_2_1_2_1_. Both space groups have eight asymmetric units per unit cell and a single polypeptide chain, labelled chain *A*, constitutes the asymmetric unit, with an estimated solvent content of 60%. The space-group assignment of *I*222 was confirmed using default settings in the program *Phaser* (McCoy *et al.*, 2007 ▶) and the structure was solved by molecular replacement and refined to a resolution of 2.50 Å. The log-likelihood gain score in *I*222 was 742, compared with a score of 86 in *I*2_1_2_1_2_1_. As will be described, *Sa*PurB forms a tetramer in the crystal structure and this rules out space group *I*2_1_2_1_2_1_. The crystallographic statistics and model geometry (Table 1 ▶) indicate that the analysis has produced an acceptable medium-resolution model. Continuous and well defined electron density was observed for the entire polypeptide except for residues 401 and 402. AMP, a product of the catalyzed reaction, was present in the crystallization conditions and well defined electron density for the ligand was observed in the active site (data not shown). Electron density compatible with PEG was located near the C-terminal segment of α7 and the C-terminal segment of α5 from a symmetry-related subunit (symmetry operation −*x* + 1/2, −*y* + 1/2, −*z* + 1/2, data not shown). In addition, a strong electron-density feature appeared in the active site which we could not explain on the basis of the known chemical components of the crystallization mixture. Different molecules were considered and refinements attempted. Our tentative interpretation is that this density represents oxalate (Fig. 2 ▶), possibly acquired as a contaminant of the PEG 200 used in crystallization. Contamination of PEG and its influence on protein crystallization has been observed previously (Jurnak, 1986 ▶). In our case, since ethylene glycol is a precursor of PEG and like PEG itself is susceptible to contamination with aldehydes and peroxides (Ray & Puvathingal, 1985 ▶), then breakdown of PEG 200 and/or the presence of ethylene glycol and subsequent oxidation may have produced oxalate.

### 3.2. Structure overview

**Figure 3:**
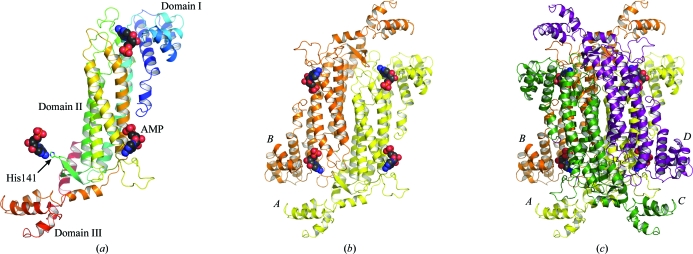
Ribbon diagrams of the *Sa*PurB subunit, the dimer and the tetramer. (*a*) A subunit is depicted and coloured from the N-terminus (blue) to the C-­terminus (red). Residues of the subunit interact with three AMP molecules, which are shown in CPK format (C, black; O, red; P, orange). (*b*) The dimer is formed from two subunits in an antiparallel arrangement. Subunit *A* is coloured yellow and subunit *B* is coloured orange. The dimer forms part of four active sites, which are marked by AMP. (*c*) The active enzyme *Sa*PurB is a tetramer. Subunit *C* is coloured green and subunit *D* is coloured purple.

The *Sa*PurB subunit can be divided into three domains that combine to form an elongated structure. Domains I and III are placed at either end of the long helical bundle formed by domain II (Fig. 3 ▶). Domain I comprises residues 1–93, which form six helices (α1–α6). Domain II, consisting of residues 94–349, forms an elongated helical bundle assembled by α7 through α15 as well as a short two-stranded antiparallel β-­sheet (β1, β2) positioned between α7 and α8. Domain III comprises five helices (α16–α20) constructed from residues 350–431. This domain carries the only unresolved portion of the structure: residues 401 and 402 that are on a loop linking α18 with α19 are disordered.

Gel filtration indicates that *Sa*PurB exists as a single species with an approximate mass of 85 kDa in solution, suggesting the presence of a dimer under the conditions used during the final stage of purification. In the crystal structure, a symmetry-related subunit (*B*) related by the crystallographic twofold axis along unit-cell edge *a* is the likely partner for such a dimeric species. The *A*–*B* interface covers an area of approximately 3380 Å^2^; almost 16% of the total surface area of the subunit. As observed in orthologues, the crystal structure reveals the formation of a tetramer (Fig. 3 ▶), which in the case of *Sa*PurB involves rotation of the *A*–*B* pair about the crystallographic axis along unit-cell edge *b* to provide the partner pair of subunits, which are labelled *C* and *D*. The *A*–*C* interface extends for approximately 1750 Å^2^ or 8% of the surface area of a subunit, whilst the smallest interface, that formed between subunits *A* and *D*, covers an area of about 1600 Å^2^ or 7.5% of the subunit surface area. Changes in the multimeric state of PurB orthologues have been reported elsewhere. The *B. subtilis* enzyme has been shown to exist largely as a dimeric species when the protein concentration is low (∼0.1 mg ml^−1^); however, as the protein concentration increases the tetrameric species becomes the most abundant form (Palenchar & Colman, 2003 ▶).

### 3.3. The active site, interactions with ligands and enzyme mechanism

The tetramer contains four active sites, each formed by regions donated from three different subunits. In the following description, *A*, *B* or *C* after the residue numbers serves to identify which subunit they belong to. We consider distances between functional groups involved in hydrogen-bonding interactions to fall within the range 2.5–3.5 Å.

**Figure 4:**
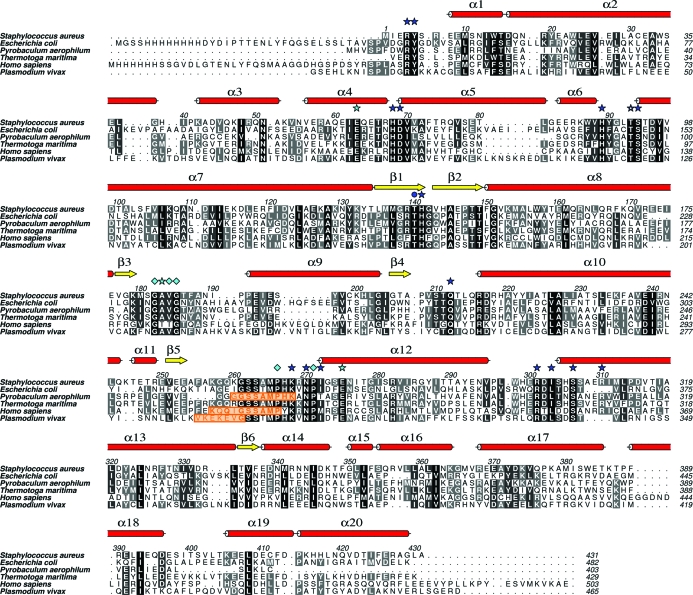
Primary and secondary structure of *Sa*PurB together with sequence alignment of selected PurB sequences. Dark blue stars indicate residues that interact with ligands in the active site; a dark blue circle identifies a threonine previously thought to be important for activity and discussed in the text. Light blue stars mark residues that help to position important side chains; light blue diamonds indicate structurally important residues. The orange boxes highlight disordered regions that correspond to the mobile active-site loop in *Sa*PurB.

A sequence alignment of PurB proteins for which structures have been determined was carried out and a subset is presented in Fig. 4 ▶ together with the secondary structure of *Sa*PurB. The PurB sequences of *T. maritima* and *B. anthracis* share 52 and 70% identity, respectively, to that of *Sa*PurB. The remainder, *L. pneumophila*, *E. coli*, *Pl. vivax*, *C. elegans* and *Py. aerophilum*, fall within a range from 20 to 33% identity. A high level of conservation in and around the ligand-binding/active site is noted, with all of the residues implicated in the enzyme mechanism being strictly conserved (Fig. 4 ▶).

**Figure 5:**
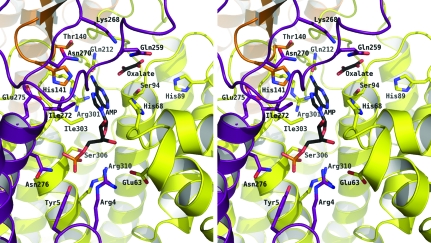
Stereoview of the active site. A molecule of AMP is bound in the active site formed by regions of three of the four subunits. There are four active sites per tetramer, with each active site formed by contributions from three of the four subunits, in this case by *A* (yellow), *B* (orange) and *D* (purple). The same colour scheme is used as in Fig. 2 ▶.

The structure of AMP in the active site of *Sa*PurB (Fig. 5 ▶) is similar to that in the *E. coli* PurB–AMP complex (Tsai *et al.*, 2007 ▶; PDB code 2ptq). The AMP phosphate accepts hydrogen bonds from Arg4*C*, Tyr5*C*, Asn276*C*, Ser306*A*, Arg310*A* and *via* a water-mediated association with Arg326*A*. The ribose O2′ donates a hydrogen bond to the Arg67*A* carbonyl and O3′ accepts hydrogen bonds from the amide of Asp69*A* and Arg4*C* NH2. The glycosidic O4′ is 3.2 Å from Asp69*A* OD1, suggesting that the latter is protonated (data not shown). The adenine N1 accepts a hydrogen bond from Thr95*A* OG1; N6 donates hydrogen bonds to His141*B* NE2 and Gln212*A* OE1, residues which are themselves positioned by hydrogen-bonding interactions. The loop Gly182-Ala183-Val184-Gly185, which is conserved in PurB sequences (Fig. 4 ▶), is positioned such that the main-chain carbonyl of Ala183 can accept a hydrogen bond from Gln212*A* NE2. His141 in *Sa*PurB corresponds to His171 in *E. coli* PurB and is the catalytic acid for the mechanism (Tsai *et al.*, 2007 ▶; Kozlov *et al.*, 2009 ▶).

The oxalate binds accepting hydrogen bonds donated from Ser94*A* OG and Gln259*A* NE2 and a water molecule that in turn interacts with the side chain of Thr95 (Fig. 2 ▶). The distance between an oxalate oxygen and Ser94 OG is 3.6 Å and this may indicate a hydrogen bond. In contrast, the oxalate is 3.4 Å distant from adenine N6 but the geometry is not optimal for hydrogen-bond formation (Leonard *et al.*, 1995 ▶). The oxalate occupies a position similar to that observed for fumarate in the active site of human PurB (PDB code 2vd6) and to that of the succinate moiety observed in the *E. coli* PurB–adenylosuccinate complex (PDB code 2ptr).

**Figure 6:**
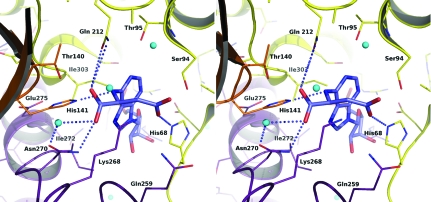
Stereoview of the adenylosuccinate model in the active site of *Sa*PurB. The colour scheme is the same as that used in Fig. 2 ▶, except that the C atoms of the ligand are shown in light blue.

A model of *Sa*PurB with adenylosuccinate was produced (Fig. 6 ▶) using the human PurB complex to aid the analysis. Two threonines, Thr93 and Thr140 in *Sa*PurB, are proposed to bind the succinyl moiety (Segall *et al.*, 2007 ▶). In *Sa*PurB, Thr93 is almost 8 Å distant from N6. There is a water-mediated link between Thr140 and Asn270 and the asparagine is the more likely residue to interact with the succinyl group. Gln212 is placed to interact with AMP N6 and a carbonyl on the modelled SAICAR–adenylosuccinate. In *Sa*PurB, Gln259 binds the oxalate, is positioned on a loop that displays elevated thermal parameters (data not shown) and could undergo a small conformational change to interact with substrate/products.

Two conserved residues, Ser94 and Ser306 in *Sa*PurB (Figs. 4 ▶ and 5 ▶), have previously attracted attention (Segall *et al.*, 2007 ▶). Ser94 is close to His68 and might influence the p*K* to favour the protonated form. In *Sa*PurB His68 ND1 accepts a hydrogen bond from the Val70 amide and thus NE2 would under normal conditions carry a proton. Our model suggests that Ser94, like Ser306, binds substrate. In *Sa*PurB Asp69 orients Arg310 so that it can in turn bind the substrate phosphate.

One side of the *Sa*PurB active site is formed by a mobile loop that links β5 and α12. The electron density for this loop is more diffuse than in other parts of the structure and the thermal parameters are higher (data not shown). In most other PurB structures this loop is completely disordered (Fig. 4 ▶). In *E. coli* PurB this loop is disordered in the apo structure but ligand binding induces a reorganization of the loop to close the ligand-binding site (Tsai *et al.*, 2007 ▶).

PurB catalyzes a β-elimination that is thought to occur *via* a general acid–base uni–bi mechanism (Bridger & Cohen, 1968 ▶; Casey & Lowenstein, 1987 ▶). Previous studies identified His141, His68 and Ser262 as important residues (Lee *et al.*, 1997 ▶, 1998 ▶, 1999 ▶; Tsai *et al.*, 2007 ▶). The conserved Ser262 of *Sa*PurB is positioned on the mobile loop near the active site and may abstract the C^β^ proton of the substrate, helping to create a carbanion intermediate. His171 is the likely proton donor to N6 to support breakage of the C—N bond. The placement of the adenine in the *Sa*PurB active site with respect to His141 suggests it is unlikely that protonation of N1 occurs during catalysis, which was a possibility presented by Tsai *et al.* (2007 ▶).

**Figure 7:**
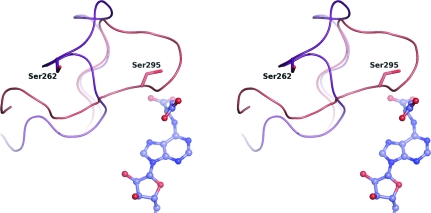
Stereoview of the different conformations displayed by the active-site loop. The *Sa*PurB structure is shown in purple and the *E. coli* enzyme structure is shown in pink. The adenylosuccinate is depicted as in Fig. 6 ▶.

His141 has been suggested to be the catalytic base (Lee *et al.*, 1997 ▶), forming a charge-relay pair with Glu275 to extract a proton from the substrate (Toth & Yeates, 2000 ▶) or alternatively Ser262 (Tsai *et al.*, 2007 ▶; corresponding to Ser295 in *E. coli*). The latter assignment was made from structural information on the apo form of *E. coli* PurB, in which the side chain of His171 (His141 in *Sa*PurB) was rotated away from the position expected for it to play a role in catalysis. In *E. coli* PurB structures the highly conserved Ser295 (Ser262 of *Sa*PurB) is close to the site of catalysis. This is not the situation in *Sa*PurB, where the conformation of the active-site loop holds Ser262 distant from the catalytic site (Fig. 7 ▶). Lys268 of *Sa*PurB, a strictly conserved residue (Fig. 4 ▶), is positioned with its NZ atom 2.8 Å from a succinyl carbonyl. Mutagenesis of the equivalent lysine in *B. subtilis* PurB decreased the enzyme activity significantly (Brosius & Colman, 2002 ▶).

Our model supports the idea that His9 of the *E. coli* enzyme, which is equivalent to *Sa*PurB His68, is not the catalytic base but rather binds and orients the substrate to interact with fumarate (Tsai *et al.*, 2007 ▶). In *Sa*PurB the oxalate is 3.8 Å distant from His68, but our model (Fig. 6 ▶) suggests that the distance would be about 3 Å in the substrate complex.

### 3.4. Dimer interface and the potential for inhibitor development

We were intrigued by two observations. Firstly, a tetramer (or dimer of dimers) is required to produce a functional enzyme since three subunits are required to contribute residues to form an active site. Secondly, there is less sequence conservation amongst the PurB residues involved in the assembly of the quaternary structure than those around the active sites. We decided to consider the dimer interface as a potential target for screening and structure-based drug-discovery methods.

Targeting transient protein–protein interactions or the interface of oligomeric assemblies by screening and structure-based methods to find small-molecule drug-like inhibitors presents a significant challenge (Yin & Hamilton, 2005 ▶). Complicating factors include the high degree of specificity and the large areas of interactions involved in complex formation. Nevertheless, PurB exists in dimeric and tetrameric forms, with enzyme activity restricted to the tetrameric form. Therefore, preventing the formation of or destabilizing the tetramer provides a route to enzyme inhibition. Our interest was furthered by the observation that there is a marked reduction in sequence conservation amongst those residues involved in the assembly of the quaternary structure.

In support of such an approach, we note that in excess of 30 point mutations have been identified that cause human adenylosuccinate lyase deficiency, several of which influence the stability of the tetramer (Spiegel *et al.*, 2006 ▶). For example, the mutation of Lys246 to glutamic acid in the human enzyme is located at the *A*–*B* or *C*–*D* subunit interface and results in a predominantly monomeric protein with negligible enzyme activity. In addition, the presence of an Arg194-to-cysteine mutation has been demonstrated to reduce the thermal stability of the *B. subtilis* enzyme tetramer, leading to significant impairment of catalytic activity (Ariyananda *et al.*, 2009 ▶).

**Figure 8:**
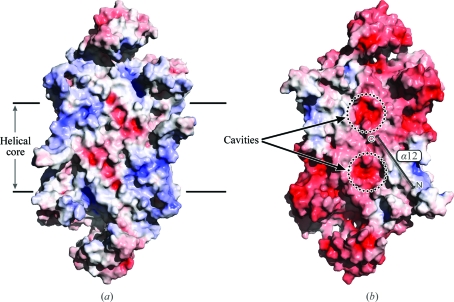
The dimer–dimer interface of (*a*) the human enzyme and (*b*) *Sa*PurB. The protein is depicted as a van der Waals surface coloured blue for basic properties, red for acidic and grey for neutral. For reference, the position of α12 in *Sa*PurB is marked.

Visualization of the electrostatic properties of the dimer–dimer interface involved in tetramer formation for both the human and *S. aureus* enzymes was achieved using the *Adaptive Poisson-Boltzman Solver* (Baker *et al.*, 2001 ▶; Fig. 8 ▶). In *Sa*PurB two acidic pockets are formed ∼13 Å from either end of the helical coil region formed by domain II of each monomer within the *A*–*B* dimer. The identical view of the human enzyme reveals a less acidic and smaller pocket. The residues forming the surface of this pocket are contributed from α10, α12 and from the loop linking α12 to α13. The symmetry of the dimer leads to a second pocket being formed 15 Å away and when the symmetry of the tetramer is considered four such pockets are created which combine to form two cavities within the core of the tetramer of approximately 13 × 10 × 10 Å in size. As mentioned perviously, targeting interface regions to disrupt oligomerization presents a significant challenge to drug discovery but this region in *Sa*PurB may be more attractive owing to the symmetry of the tetramer. The effect of a small molecule targeted to the pocket on the surface of one subunit could be amplified by the proximity of four such pockets, offering the potential to exploit multivalent ligands.

## 4. Conclusions

The *Sa*PurB structure displays a conserved fold and mode of oligomerization compared with orthologous enzymes. The active sites are highly conserved between species and this extends to the presence of a flexible loop at the active site. The *Sa*PurB structure contained AMP and a likely contaminant, oxalate, in the active site. The high degree of conservation in the PurB active sites would render it difficult to identify a potent species-specific inhibitor of value in the development of new therapeutic agents. In contrast, the dimer interface of PurB from *S. aureus* appears to be sufficiently distinct from that observed for the human orthologue that it may represent a potential target for which inhibitors might be developed. However, we note the not inconsiderable difficulties in adopting such an approach.

## Supplementary Material

PDB reference: adenylo­succinate lyase, 2x75

## Figures and Tables

**Table 1:** Crystallographic statistics Values in parentheses are for the highest resolution shell.

Space group	*I*222
Unit-cell parameters (Å)	*a* = 81.6, *b* = 121.3, *c* = 105.7
Resolution range (Å)	97–2.5 (2.64–2.5)
No. of unique reflections	17795
Completeness	96.9 (98.7)
Redundancy	5.3 (5.3)
Wilson *B* factor (Å^2^)	56.8
〈*I*/σ(*I*)〉	14.3 (2.6)
*R*_merge_†	4.7 (49.2)
No. of protein residues	428
No. of water molecules	70
No. of AMP molecules	1
No. of oxalate molecules	1
No. of chloride ions	1
*R*_work_‡/*R*_free_§ (%)	21.7/28.4
R.m.s.d. from ideal values	
Bond lengths (Å)	0.010
Angles (°)	1.160
Average *B* factors (Å^2^)	
Overall	68.6
Side chain	69.1
Main chain	68.0
AMP	59.9
Oxalate	73.6
PEG	61.4
Chloride	61.0
Ramachandran plot analysis	
Favourable	383 [92.5%]
Additionally allowed	7 [6.5%]
Outliers	4 [1%]

† *R*_merge_ = 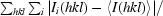 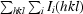, where *I_i_*(*hkl*) is the intensity of the *i*th measurement of reflection *hkl* and 〈*I*(*hkl*)〉 is the mean value of *I_i_*(*hkl*) for all *i* measurements.

‡ *R*_work_ = 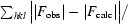 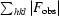, where *F*_obs_ is the observed structure-factor amplitude and *F*_calc_ is the structure-factor amplitude calculated from the model.

§ *R*_free_ is the same as *R*_work_ except calculated with a subset (5%) of data that were excluded from refinement calculations.
